# Strength Development and Microscopic Characterization of Slag-like Powder Materials Activated by Sodium Carbonate and Sodium Hydroxide

**DOI:** 10.3390/ma18102313

**Published:** 2025-05-15

**Authors:** Donghui Li, Wenzhong Zheng, Ying Wang

**Affiliations:** 1 School of Civil Engineering, Harbin Institute of Technology, Harbin 150090, China; tumu_donghui2016@163.com (D.L.); wangying888@hit.edu.cn (Y.W.); 2 Key Lab of Structures Dynamic Behaviour and Control of the Ministry of Education, Harbin Institute of Technology, Harbin 150090, China; 3 Key Lab of Smart Prevention and Mitigation of Civil Engineering Disasters of the Ministry of Industry and Information Technology, Harbin Institute of Technology, Harbin 150090, China

**Keywords:** alkali-activated slag-like powder materials, sodium carbonate, sodium hydroxide, strength development, microstructure

## Abstract

Alkali-activated slag-like powder (AASP) materials are a novel type of binder prepared by activating slag-like powder (SP) with alkaline activators, providing a sustainable alternative to traditional cement for construction in remote mountainous regions, as well as on islands and reefs far from the inland, reducing transportation costs, shortening construction timelines, and minimizing energy consumption. SP is locally produced from siliceous and calcareous materials through calcining, water quenching, and grinding, exhibiting reactivity similar to that of ground granulated blast-furnace slag. In this study, siliceous sand and ground calcium carbonate powder were utilized to produce SP, with sodium carbonate (Na_2_CO_3_), sodium hydroxide (NaOH), and their mixture serving as activators. The results indicated that the Ca/Si ratio in SP, along with the dosage of Na_2_CO_3_ (*D*_sc_) and Na_2_O content (*N*_c_) in the activator, significantly affected the compressive strength of AASP materials at both early and late stages. The 28-day compressive strength reached up to 78.95 MPa, comparable to that of alkali-activated slag (AAS) materials. The optimum mix ratio for Na_2_CO_3_-NaOH based AASP materials was also determined to be 80% *D*_sc_ and 8% *N*_c_ (C8N2-8). Microscopic analyses were employed to investigate the changes in the macroscopic properties of AASP materials driven by hydration products, chemical group composition, and microstructure.

## 1. Introduction

In remote mountainous regions, as well as on islands and reefs far from the inland, geographic isolation presents considerable challenges to the transportation of construction materials for infrastructure development [1,2]. The rugged mountain terrain, along with the long distances from inland areas, not only increase transportation costs, but also extend construction timelines and elevate energy consumption, all of which negatively affect the overall project efficiency [3,4,5]. Faced with this issue, scholars have devoted their efforts to utilizing local resources, such as rocks [6,7], pebbles [8], sand [7,8], and soil [9] from mountainous regions, as well as seawater [10,11], sea sand [12,13], and waste coral [5,14,15] from or around islands and reefs. However, while their research has employed these locally sourced materials as fillers in components or aggregates for concrete, no investigation has yet been conducted into their potential use for the preparation of cementitious substances as an alternative to cement.

Ground granulated blast-furnace slag (abbreviated as slag), a by-product of ironmaking industry, is primarily composed of CaO, SiO_2_, and Al_2_O_3_ [16,17]. Following melting, rapidly cooling, and grinding, slag demonstrates latent hydraulic properties and facilitates its reaction with alkaline activators, resulting in the formation of a binder with cement-like characteristics, known as alkali-activated slag (AAS) materials [18]. The analysis of the main chemical components in slag reveals that CaO and Al_2_O_3_ enhance the reactivity of slag and improve the early-age strength of the paste [18,19,20]. Additionally, CaO provides nucleation sites that trigger the formation of calcium silicate hydrate (C-S-H) gel [21,22,23,24], while SiO_2_ supplies [SiO_4_]^4−^ ions and establishes the framework of the C-S-H gel [25,26]. Thus, both CaO and SiO_2_ are crucial for slag to generate AAS materials. The production process of AAS materials offers valuable insights and inspiration for the development of a sustainable binder utilizing locally available materials. Siliceous materials, including siliceous mountain sand and siliceous sea sand (mainly SiO_2_), were mixed with calcareous materials, such as calcareous mountain sand, calcareous sea sand, and waste coral (mainly CaCO_3_), in specific ratios. The mixture was heated in a medium-frequency induction furnace until it melted, then water quenched in a pool and ground in a ball mill to yield slag-like powder (SP). The SP was subsequently activated with appropriate activators, leading to the production of alkali-activated slag-like powder (AASP) materials.

AAS materials are a type of alkali-activated materials (AAMs), which are generated by the reactions between precursors and alkaline activators [27,28,29]. The precursors can be classified into two categories based on their CaO content [29,30]. The first category comprises high-calcium materials, including slag, Class C fly ash (HCFA), and steel slag. The AAMs derived from these materials are referred to as high-calcium systems, with calcium aluminosilicate hydrate (C-(A)-S-H) gel as the primary hydration product [31,32]. The second category consists of low-calcium materials, such as Class F fly ash (LCFA), metakaolin (MK), and volcanic ash. The AAMs produced from these materials are known as low-calcium systems, which form sodium aluminosilicate hydrate (N-A-S-H) gel as the main reaction product [32,33]. There are several differences between high-calcium and low-calcium systems in terms of reaction conditions, strength development, high temperature resistance, and chloride permeability. Regarding the reaction conditions, high-calcium systems generally react at room temperature, while low-calcium systems require higher temperatures due to the lower reactivity of low-calcium materials [34] and the more complex reaction mechanisms involved [35]. Singh et al. [28] concluded that the optimal curing temperature range for low-calcium systems lay between 40 °C and 85 °C, while Lemougna et al. [34] suggested that a curing temperature range of 50 °C to 100 °C was necessary for volcanic ash to effectively prepare AAMs. Additionally, Lee and Lee [36] identified 60 °C as the lowest temperature at which LCFA-based AAMs could achieve a high strength. With respect to strength development, high-calcium systems exhibit a more rapid rate of strength gain [27,28]. Bai et al. [37] investigated the effect of steel slag on the early-age strength of MK-based AAMs, and found that a 10% inclusion of steel slag increased the 8-h compressive strength by 450%. Zhu et al. [38] reported that as the LCFA content increased, the slag-based AAMs experienced a substantial reduction in early-age strength, with a decline of up to 71.4% when the LCFA content reached 80%. Regarding high temperature resistance, low-calcium systems offer greater advantages due to the generated N-A-S-H gel, which holds a small amount of chemically bound water [32], and its porous structure efficiently mitigates vapor pressure [39]. Aziz et al. [40] studied the compressive strength of slag-based AAMs after elevated temperatures, and observed a consistent decline in strength as the temperature rose from 200 °C to 800 °C, with the residual strength at 800 °C dropping to just 9.4% of the strength at ambient temperature. In contrast, Hassan et al. [41] found that LCFA-based AAMs retained 72.7% of the compressive strength at 25 °C after exposure to 800 °C. In terms of chloride permeability, the C-(A)-S-H gel formed in high-calcium systems contributes to a matrix with low porosity and high tortuosity [31,42,43], while the N-A-S-H gel generated in low-calcium systems possesses high specific surface area, which enhances its capability to bind chloride ions [31,44,45]. As a result, both systems exhibit strong resistance to chloride penetration. However, different conclusions were reached when comparing their chloride permeability. Ismail et al. [44] demonstrated through experimental studies that slag-based AAMs exhibited improved durability in chloride environments compared to LCFA-based AAMs. On the other hand, Kupwade and Allouche [46] argued that LCFA-based AAMs displayed superior resistance to chloride penetration in comparison with HCFA-based AAMs. From the above, it is evident that the CaO content in the precursors has a significant impact on the performance of AAMs.

Alkaline activators create an alkaline environment that facilitates the precursor dissolution and triggers alkali-activation reactions [47,48,49]. The type and dosage of the activator are crucial to both the hydration process and the properties of the resulting paste, as they regulate the pH of the solution and introduce various ions [50,51]. There are three main types of alkaline activators: (1) alkali silicates, represented by liquid sodium silicate (LSS); (2) alkali hydroxides, represented by sodium hydroxide (NaOH); and (3) alkali carbonates, represented by sodium carbonate (Na_2_CO_3_) [30,52]. Potassium-based compounds are restricted in alkali-activation applications due to their high cost and difficulty in accessibility [51,53]. LSS is considered the most effective activator due to the additional [SiO_4_]^4−^ ions it provides for C-S-H gel formation [54,55]. However, some drawbacks of AAMs activated by LSS, such as rapid setting [48,56] and excessive shrinkage [48,57], significantly limit their practical applications. Jiao [58] measured the initial and final setting times of LSS-activated slag, which ranged from 17 to 38 min and 25 to 48 min, respectively. Atis et al. [59] observed that the shrinkage of AAMs activated by LSS was three times greater than that of cement mortar, whereas the shrinkage of AAMs activated by Na_2_CO_3_ was either lower or comparable to cement mortar. NaOH serves as the most widely used hydroxide activator, exhibiting stronger alkalinity compared to LSS and promoting the dissolution of precursors [60]. Consequently, the NaOH-activated slag shows high early-age strength [48], and Jiao et al. [57] reported a compressive strength of 20.3 MPa at 1 day when the Na_2_O content (*N*_c_) was 6%. The strength at 6% *N*_c_ was higher than that at both 4% and 8% *N*_c_ values. They suggested that as the *N*_c_ increased, the concentration of OH^−^ ions in the solution rose, leading to an increase in the strength. However, excess alkali did not react with the slag and negatively impacted the strength. Despite this, the late-age strength of NaOH-activated slag is lower than that of AAMs activated by LSS and Na_2_CO_3_, due to the C-S-H gel displaying the lowest polymerization degree [61]. Na_2_CO_3_ is the only activator among the three that can be sourced as a natural mineral resource, with nearly half of its global production derived from geological mining [49,52]. As a result, it offers notable advantages in energy conservation and environmental sustainability [62,63]. However, due to its low alkalinity, it is limited to activating high-calcium materials [30,47,51]. The ${CO}_{3}^{2-}$ ions in Na_2_CO_3_ preferentially react with Ca^2+^ ions to form insoluble carbonates prior to the C-S-H gel formation, leading to a slow setting rate and low early-age strength of the resulting AAMs [50,64]. Li et al. [56] activated slag with Na_2_CO_3_, and measured that the initial and final setting times were 300 min and 5 days, respectively. Kovtun et al. [65] observed that Na_2_CO_3_-activated slag remained soft at 1 day, and Akturk et al. [62] obtained a 3-day compressive strength of only 0.6 MPa. However, as the ${CO}_{3}^{2-}$ ions are consumed, the pH of the solution increases, accelerating the reaction and enhancing the late-age strength [48,50,66]. Furthermore, the insoluble carbonates, such as calcite, exert a positive effect on pore filling and refinement, contributing to the improvement of the late-age strength [67]. Abdalqader et al. [63] used Na_2_CO_3_ as an activator, and the compressive strength of the slag-based AAMs at 28 days exceeded 70 MPa. The increase in Na_2_CO_3_ content from 5% to 10% enhanced the strength at all ages. To overcome the limitations of using a single activator, many researchers have employed a mixture of Na_2_CO_3_ and NaOH as activators, and the produced AAMs exhibited excellent mechanical properties [57,62,65]. Dai et al. [68] added 20% NaOH to Na_2_CO_3_, and found that the final setting time of the slag-based AAMs decreased from 1320 to 300 min, the 48-h compressive strength increased from 0 to 21.5 MPa, and the 28-day strength rose from 54 to 70.2 MPa. Meanwhile, the matrix became more compact and uniform. Li and Sun [69], Akturk et al. [62], and Jiao et al. [57] jointly confirmed that the effect of mixed activation with Na_2_CO_3_ and NaOH was superior to activation by Na_2_CO_3_ or NaOH alone. Kovtun et al. [65] proposed that the optimum ratio of Na_2_CO_3_ to NaOH was 1:1.

To summarize, the CaO content of the precursor, along with the type and dosage of the activator, plays a crucial role in the properties of AAMs. Therefore, this experimental research was conducted to investigate the effects of the Ca/Si ratio (molar ratio of CaO to SiO_2_) in SP (1.25, 1.0, 0.75, 0.5, and 0.25), the activator type (Na_2_CO_3_, NaOH, and a mixture of Na_2_CO_3_ and NaOH), and the activator dosage (2%, 4%, 6%, and 8%) on the mechanical properties of AASP materials, with the goal of determining the optimum mix ratio and offering guidance for engineering applications. Furthermore, the selected samples were analyzed using X-ray diffraction (XRD), Fourier transform infrared (FT-IR) spectroscopy, and scanning electron microscopy with energy dispersive spectroscopy (SEM-EDS) to make a corresponding explanation for the variation in compressive strength from a microscopic perspective.

## 2. Experimental Program

### 2.1. Materials

Given that particle sizes ranging from 0.09 to 0.45 mm are known to be easily meltable [70], the selected siliceous sand, with a particle size of 0.25 mm and a composition of 91.59% SiO_2_ and 0.15% CaO, was obtained from Pengjian Mineral Processing Plant, Hebei, China. Ground calcium carbonate powder is produced by crushing and grinding natural calcite and limestone, with CaCO_3_ as its main chemical component. The ground calcium carbonate powder (99.52% CaCO_3_ purity) with a particle size of 37 μm, obtained from Xinyang Ltd., Henan, China, was used in the experiment.

The alkaline activator was prepared using Na_2_CO_3_, NaOH, or their mixture. Powdery anhydrous Na_2_CO_3_ (99.8% analytical purity) was sourced from Zhiyuan Reagent Ltd., Tianjin, China, while beaded NaOH (96% analytical purity) was supplied by Continental Chemical Reagent Factory, Tianjin, China.

### 2.2. SP Preparation

**Figure 1:**
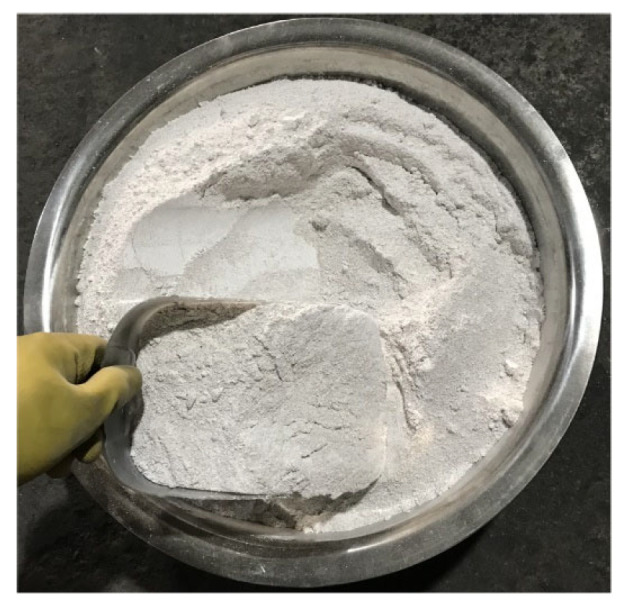
Mixed raw materials.

**Figure 2:**
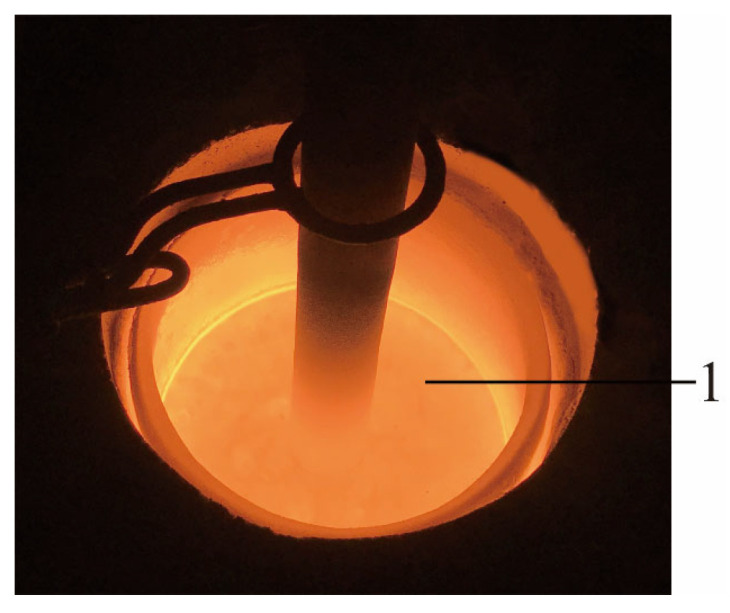
Melting process; 1—molten raw materials.

**Figure 3:**
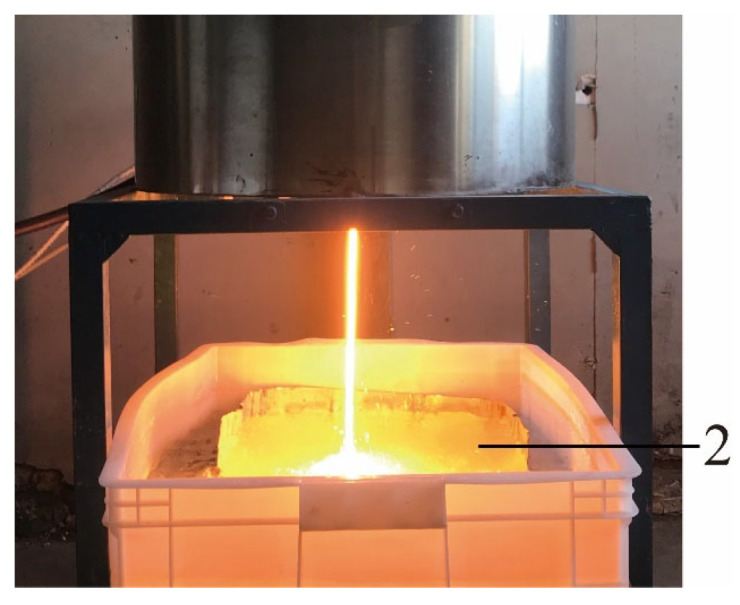
Water quenching process; 2—water quenching pool.

**Figure 4:**
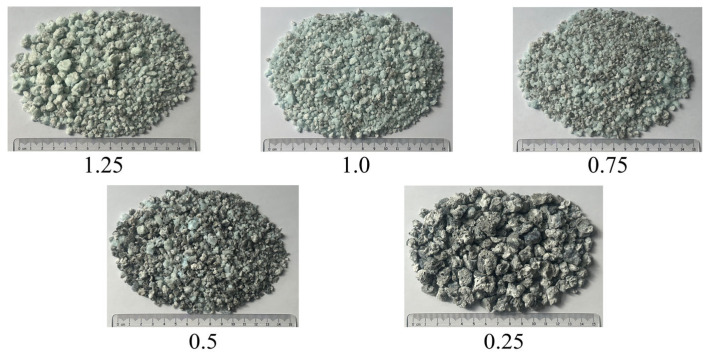
Slag-like particles with different Ca/Si ratios.

**Figure 5:**
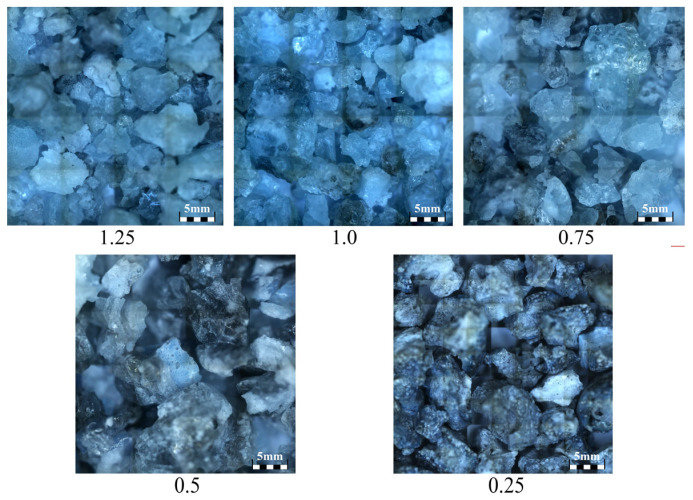
Micromorphology of slag-like particles (×69).

Siliceous sand and ground calcium carbonate powder were used to produce SP. First, as depicted in Figure 1, they were weighed and mixed according to the proportions listed in Table 1. Second, as illustrated in Figure 2, the mixed raw materials were calcined in a medium-frequency induction furnace until they melted at approximately 1700 °C. Third, as shown in Figure 3, the molten materials were water quenched to transform the crystals into vitreous phases, and the generated slag-like particles are illustrated in Figure 4. Significant differences were observed among the slag-like particles with various Ca/Si ratios. When the Ca/Si ratio was 1.25, the particles exhibited a light green color and an uneven particle size. With the Ca/Si ratio decreasing from 1.0 to 0.5, gray-black particles emerged, and their proportion gradually increased, with the particle size being small and uniform. When the Ca/Si ratio reduced to 0.25, the particles became entirely gray-black, with a large and consistent particle size. As presented in Figure 5, the micromorphology of slag-like particles, captured with a super-depth-of-field microscope, revealed a large number of irregularly shaped vitreous particles. The vitreous body content progressively diminished with the reduction in the Ca/Si ratio, indicating a decline in the activity of SP [71].

According to the GB/T 18046-2017 standard [72], the slag-like particles were ground to a specific surface area of about 400 m^2^/kg (determined by the BET method following the GB/T 19587-2017 standard [73]) to satisfy the reactivity requirement of S95-grade slag. Due to the differences in grindability, the grinding durations were experimentally evaluated: particles with Ca/Si ratios of 1.25 and 1.0 were ground for 100 min, those with Ca/Si ratios of 0.75 and 0.5 for 80 min, and those with a Ca/Si ratio of 0.25 for 60 min. As listed in Table 2, X-ray fluorescence (XRF) spectrometry was used to determine the main chemical composition of SP, with the derived Ca/Si ratios also presented. The maximum deviation was 1.258 for the actual Ca/Si ratio compared to the expected value of 1.25, with a negligible difference of 0.008.

**Figure 6:**
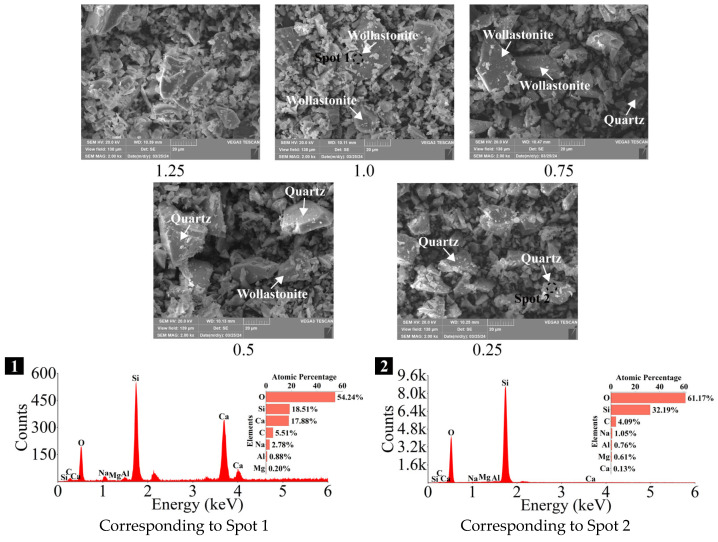
SEM-EDS analyses of SP with varying Ca/Si ratios (×2k).

As depicted in Figure 6, SEM was utilized to observe the microstructure of SP, while EDS was employed to help identify and differentiate the various crystal phases present. There were no significant distinctions in the microstructures of SP with different Ca/Si ratios, and all of them exhibited angular and irregular shapes, with particles of varying sizes. However, the crystal phases varied among the samples, with wollastonite (CaSiO_3_) and quartz (SiO_2_) identified through EDS analyses. In the SP with a Ca/Si ratio of 1.25 (denoted as 1.25 SP hereafter), no crystal phases were detected, indicating that it consisted of vitreous bodies, which exhibited the highest reactivity [74]. A single crystal phase of wollastonite (CaSiO_3_) was observed in the 1.0 SP. In the 0.75 SP and 0.5 SP, two crystal phases were detected: wollastonite and quartz (SiO_2_). The 0.25 SP, however, contained only a single crystal phase of quartz.

### 2.3. Mix Ratio Design

Five types of SP were used as precursors, and the water to SP ratio by mass was constant at 0.35. The composition and alkalinity of the activator were represented by *D*_sc_ (dosage of Na_2_CO_3_, wt.% of the activator) and *N*_c_ (Na_2_O content, wt.% of SP), respectively. The variation of *D*_sc_ was from 0% to 100%. When the *D*_sc_ was 0% or 100%, the activator was either NaOH or Na_2_CO_3_ alone, respectively. However, at 20% or 80% *D*_sc_, it was a mixture of 20% Na_2_CO_3_ and 80% NaOH, or 80% Na_2_CO_3_ and 20% NaOH, respectively. The *N*_c_ ranged from 2% to 8% with an interval of 2%, and the alkalinity of the activator with 2% *N*_c_ was too low, causing the specimens prepared using 0.5 SP and 0.25 SP to fail to set at 3 days. Consequently, a total of 72 mixes were employed in this experiment, including 16 sets for 1.25 SP, 1.0 SP, and 0.75 SP, and 12 sets for 0.5 SP and 0.25 SP, as detailed in Table 3.

### 2.4. Methodology

#### 2.4.1. Compressive Strength Determination

**Figure 7:**
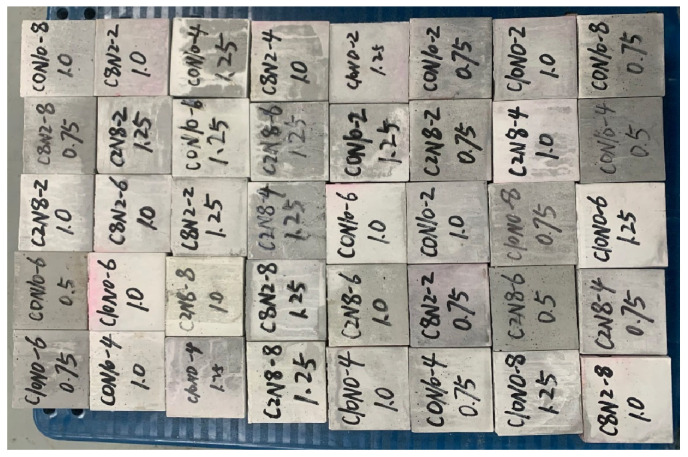
Compression test specimens.

The activator was formulated one day in advance following the mix ratios [15]. The SP and activator were added to the cement agitator in sequence, and the mixing process comprised two stages: initially slow stirring followed by fast stirring, with each lasting 90 s. Based on the JGJ/T 70-2009 standard [75], the mixture was placed into 70.7 mm cube molds and vibrated for 60 s to ensure uniform compaction. The specimens were covered with a plastic film and cured under standard conditions (21 °C, 96% RH), then demolded after 3 days, as shown in Figure 7. The compressive strength was measured at 3, 7, 28, and 90 days (with 3 and 7 days for early-age strength, while 28 and 90 days for late-age strength [59,76]) using a WHY-1000 compression tester (Hualong, Shanghai, China) with a loading rate of 1.5 kN/s. The reported strength was the average of three specimens.

#### 2.4.2. Microscopic Analyses

Microscopic analyses were performed on the 28-day samples, taken from the center of the failure surface of specimens during the compressive strength determination [77]. The samples were placed in a vacuum freezing dryer for 3 days to remove moisture and stop further hydration. The samples for XRD and FT-IR analyses were ground into powder until passing a 45-μm sieve, while those for SEM-EDS analyses were broken into about 5-mm pieces with flat surfaces.

**Figure 8:**
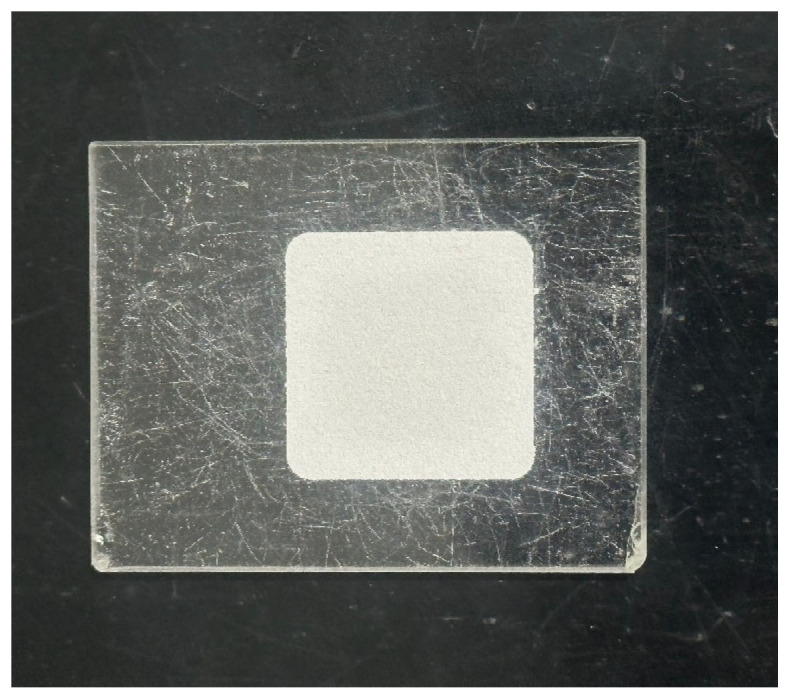
XRD analysis sample.

**Figure 9:**
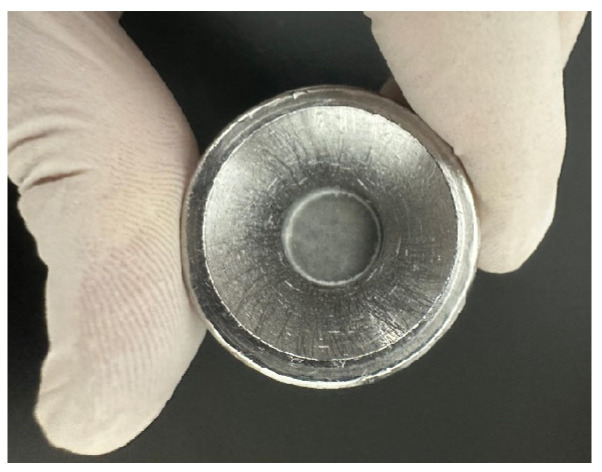
FT-IR analysis sample.

As presented in Figure 8, the powder was placed into the sample tank, and a cover plate was used to evenly distribute and compact it. An X’Pert3 Powder X-ray diffractometer (PANalytical, Almelo, the Netherlands) was employed for XRD analysis, with a scanning angle range of 5–80° and a step size of 0.02°. As shown in Figure 9, the sample was mixed with KBr in a mass ratio of 1:100 in an agate mortar, and a tablet press was used to apply a pressure of 15 MPa on the mixture to produce the flake sample. The FTIR-650 spectrometer (Gangdong, Tianjin, China) was used for FT-IR analysis, with a scanning wavenumber range of 4000–400 cm^−1^ and a resolution of 4 cm^−1^. The samples for SEM-EDS were attached to an aluminum sheet using carbon tape and then gold sputter-coated to improve conductivity. The analyses were conducted using a VEGA3 XMU SEM (Tescan, Brno, Czech Republic) in secondary electron (SE) mode.

## 3. Results and Discussion

### 3.1. Compressive Strength Development

**Figure 10:**
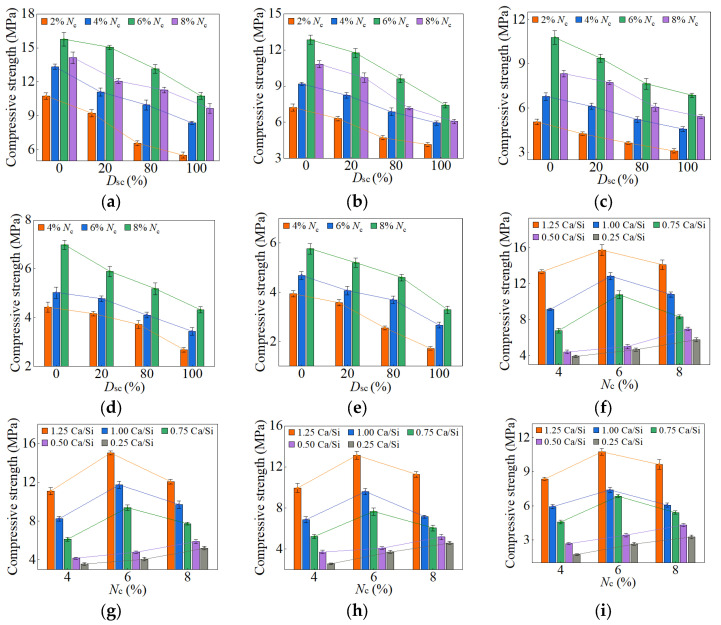
3-day compressive strength of AASP materials: (**a**) 1.25 M; (**b**) 1.0 M; (**c**) 0.75 M; (**d**) 0.5 M; (**e**) 0.25 M; (**f**) variation with Ca/Si ratio (*D*_sc_ = 0%); (**g**) variation with Ca/Si ratio (*D*_sc_ = 20%); (**h**) variation with Ca/Si ratio (*D*_sc_ = 80%); (**i**) variation with Ca/Si ratio (*D*_sc_ = 100%). Note: AASP materials produced from 1.25 SP, 1.0 SP, 0.75 SP, 0.5 SP, and 0.25 SP were designated as 1.25 M, 1.0 M, 0.75 M, 0.5 M, and 0.25 M, respectively.

**Figure 11:**
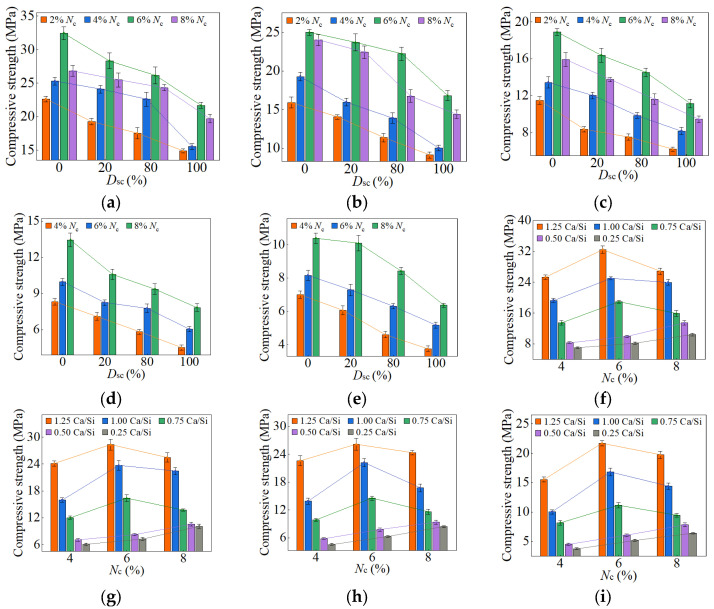
7-day compressive strength of AASP materials: (**a**) 1.25 M; (**b**) 1.0 M; (**c**) 0.75 M; (**d**) 0.5 M; (**e**) 0.25 M; (**f**) variation with Ca/Si ratio (*D*_sc_ = 0%); (**g**) variation with Ca/Si ratio (*D*_sc_ = 20%); (**h**) variation with Ca/Si ratio (*D*_sc_ = 80%); (**i**) variation with Ca/Si ratio (*D*_sc_ = 100%).

**Figure 12:**
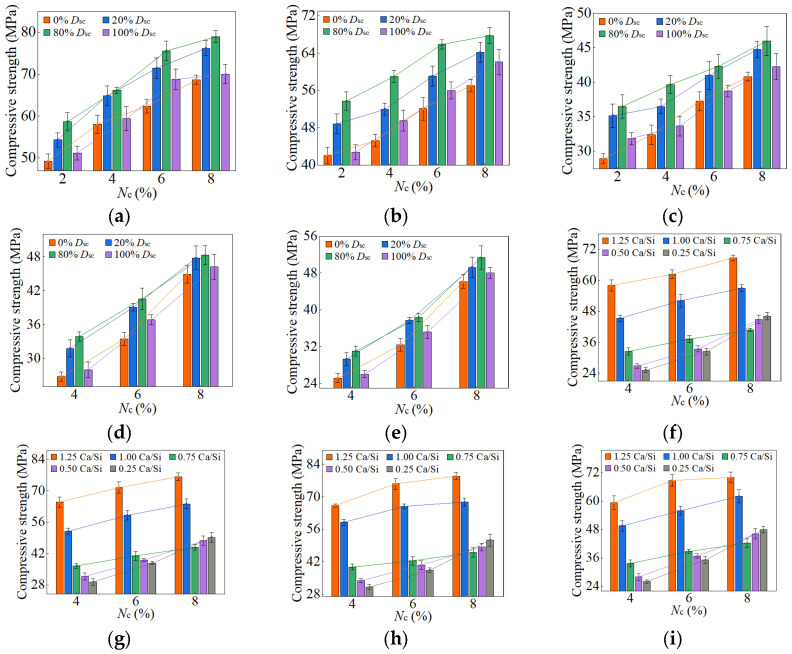
28-day compressive strength of AASP materials: (**a**) 1.25 M; (**b**) 1.0 M; (**c**) 0.75 M; (**d**) 0.5 M; (**e**) 0.25 M; (**f**) variation with Ca/Si ratio (*D*_sc_ = 0%); (**g**) variation with Ca/Si ratio (*D*_sc_ = 20%); (**h**) variation with Ca/Si ratio (*D*_sc_ = 80%); (**i**) variation with Ca/Si ratio (*D*_sc_ = 100%).

**Figure 13:**
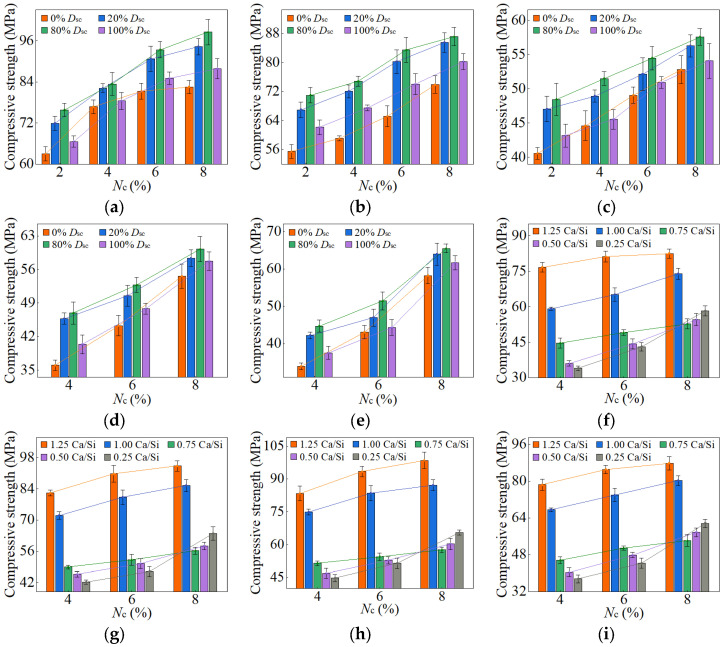
90-day compressive strength of AASP materials: (**a**) 1.25 M; (**b**) 1.0 M; (**c**) 0.75 M; (**d**) 0.5 M; (**e**) 0.25 M; (**f**) variation with Ca/Si ratio (*D*_sc_ = 0%); (**g**) variation with Ca/Si ratio (*D*_sc_ = 20%); (**h**) variation with Ca/Si ratio (*D*_sc_ = 80%); (**i**) variation with Ca/Si ratio (*D*_sc_ = 100%).

When the *D*_sc_ ranged from 0% to 100%, and the *N*_c_ fell between 2% and 8%, the compressive strengths of AASP materials at 3, 7, 28, and 90 days are presented in Figure 10, Figure 11, Figure 12 and Figure 13, respectively. Figure 10, Figure 11, Figure 12 and Figure 13 (a–e) illustrate the effects of *D*_sc_ and *N*_c_ on the compressive strength for each Ca/Si ratio, while Figure 10, Figure 11, Figure 12 and Figure 13 (f–i) depict the influences of *N*_c_ and Ca/Si ratio on the compressive strength for each *D*_sc_.

As presented in these figures, the compressive strengths varied from 1.71 to 15.76 MPa at 3 days, from 3.75 to 32.47 MPa at 7 days, from 25.17 to 78.95 MPa at 28 days, and from 33.96 to 98.52 MPa at 90 days, indicating a continuous increase in strength with the extension of curing age, and the strength development rate could also be obtained, and is depicted in Table 4. The 3-day and 7-day strengths were around 7–29% and 13–52%, respectively, with respect to the 28-day strength. And the strength at 28 days was about 69–83% of that at 90 days. Akturk et al. [62] acquired the compressive strength of Na_2_CO_3_-NaOH based AAS materials at different ages in the experiment, and the strength development rate was calculated and presented in Table 4. Similarly, the measured strength development rates of LSS-activated SP materials and ordinary Portland cement (OPC) paste in the previous experiment [78] were also presented in Table 4. It showed that the strength development of both AASP and AAS materials at 3 days was slow, with a rate lower than that of LSS-activated SP materials. It was ascribed to the activators, which were unable to provide [SiO_4_]^4−^, leading to a longer time required for OH^−^ in the solution to break the Si-O bonds in SP or slag and release [SiO_4_]^4−^ [54]. Furthermore, if the activator ionized to generate ${CO}_{3}^{2-}$ ions, they preferentially bound with Ca^2+^ to form insoluble carbonates, resulting in a slow development of 3-day strength. At 7 days, the strength development rate of AASP materials reached 52%, which was 35% lower than that of AAS materials. It was because of the 1% Al_2_O_3_ content in SP, while the Al_2_O_3_ content in slag was in the range of 5–33% [79], which enhanced the reactivity of the slag and accelerated the early-age strength development of the paste [18,19,20]. The strength development rate of AASP materials could reach up to 83%, equivalent to that of OPC, indicating that the strength basically achieved stable by 28 days. Li and Sun [69] produced AAS materials with Na_2_CO_3_-NaOH and obtained a 28-day compressive strength ranging from 31.6 MPa to 63.1 MPa, suggesting that AASP materials exhibited a strength level similar to AAS materials.

#### 3.1.1. Effect of *D*_sc_ on Strength

Figure 10 and Figure 11 and Table 5 illustrate the effects of *D*_sc_ on the early-age compressive strengths and strength development rates of the pastes, respectively. When the *D*_sc_ was 0% (i.e., activated by NaOH), the 3-day compressive strength ranged from 3.93 to 15.76 MPa, and the strength development rate was in the range of 12–29%; the 7-day strength ranged from 6.99 to 32.47 MPa, and the strength development rate was in the range of 23–52%. With the increase of *D*_sc_, the early-age compressive strengths and strength development rates gradually decreased. When the *D*_sc_ reached 100% (i.e., activated by Na_2_CO_3_), the 3-day compressive strength varied from 1.71 to 10.75 MPa, and the strength development rate fell between 7% and 18%; the 7-day strength varied from 3.75 to 21.69 MPa, and the strength development rate fell between 13% and 32%. As presented in Equation (1), Na^+^ and ${CO}_{3}^{2-}$ ions were produced by the ionization of Na_2_CO_3_, and they preferentially reacted with the Ca^2+^ ions generated by the dissolution of SP to form insoluble carbonates: calcite (CaCO_3_) and gaylussite (Na_2_Ca(CO_3_)_2_·5H_2_O). An increase in *D*_sc_ correspondingly raised the concentrations of ${CO}_{3}^{2-}$ and Na^+^ ions, which resulted in more Ca^2+^ consumption and reduced C-S-H gel formation, yielding adverse effects on the early-age strength development [62,69].


(1) $${Na}_{2}{CO}_{3}\to 2{Na}^{+}+{CO}_{3}^{2-}$$

As depicted in Figure 12 and Figure 13, the late-age compressive strengths of the five types of pastes showed a trend of initially increasing and subsequently decreasing with the rise of *D*_sc_. When the *D*_sc_ ranged from 0% to 80%, the late-age compressive strength gradually increased. However, as the *D*_sc_ further reached 100%, the strength declined accordingly. Therefore, the sequence of late-age compressive strengths for the pastes with different *D*_sc_ values was determined as follows: C0N10 < C10N0 < C2N8 < C8N2, indicating that the paste activated by NaOH had the lowest strength, while the paste activated by a mixture of 80% Na_2_CO_3_ and 20% NaOH exhibited the highest strength. Both the amount and polymerization degree of the generated C-S-H gel influenced the late-age strength development, and positively correlated with it [76,80]. In the paste activated by NaOH, the generated C-S-H exhibited a high content of Q^2^ silicon, but almost no Q^3^ silicon, suggesting a long linear chain structure with a low polymerization degree, which resulted in the lowest late-age strength [61,79]. In contrast, the C-S-H gel formed in the paste activated by Na_2_CO_3_ showed a high content of Q^3^ silicon, but a low content of Q^2^ silicon, indicating a dense structure with a high polymerization degree, which led to higher late-age strength [55,61,65]. When activated with a mixture of Na_2_CO_3_ and NaOH, the presence of NaOH increased the OH^−^ concentration and promoted the SP dissolution, thereby enhancing the C-S-H formation and improving the late-age strength [58]; the presence of Na_2_CO_3_ elevated the polymerization degree of the C-S-H gel, thereby increasing the late-age strength. Furthermore, Cai and Ye [67] proposed that insoluble carbonates, such as calcite, exerted a positive effect on pore refinement and strength development, a perspective also supported by Zhu et al. [81]. This provided an explanation for the higher strength of the paste with 80% *D*_sc_ compared to that with 20% *D*_sc_. To sum up, the mixed activation with Na_2_CO_3_ and NaOH was more effective than activation by Na_2_CO_3_ or NaOH alone [53,65,69].

#### 3.1.2. Effect of *N*_c_ on Strength

The variation in compressive strength with the *N*_c_ was associated with the Ca/Si ratio of SP. When the Ca/Si ratio was 1.25, 1.0, and 0.75, the early-age strengths first rose and then decreased as *N*_c_ increased from 2% to 8%, with the inflection point at 6% *N*_c_. The *N*_c_ directly determined the concentration of OH^−^ ions; thus, a higher *N*_c_ promoted the hydration reaction rate, resulting in more C-S-H formation and increased early-age strength [82]. However, 8% *N*_c_ led to an excessively rapid reaction, forming a dense “reaction ring” of hydration products around the SP, which slowed down the reaction and diminished the early-age strength [57,83]. With the curing age extending, the OH^−^ ions progressively diffused through the “reaction ring”, facilitating the continued dissolution of unreacted SP and the formation of C-S-H gel, thereby increasing the strength [79]. As a result, the late-age compressive strengths of the pastes rose steadily with *N*_c_ varying from 2% to 8%.

When the Ca/Si ratio was 0.5 and 0.25, the corresponding pastes exhibited a continuous increase in both early-age and late-age compressive strengths with *N*_c_ ranging from 4% to 8%. The CaO content in 0.5 SP and 0.25 SP was low, resulting in fewer Ca^2+^ ions being released into the solution in the dissolving process. Therefore, the Ca^2+^ concentration controlled the reaction rate, inhibiting the formation of “reaction rings” and preventing a decrease in strength.

#### 3.1.3. Effect of Ca/Si Ratio on Strength

When the *D*_sc_ ranged from 0% to 100%, and the *N*_c_ was in the range of 4–8%, the decrease in the Ca/Si ratio resulted in a reduction in early-age compressive strength. A lower Ca/Si ratio corresponded to a lower CaO content in SP, which reduced the release of Ca^2+^ ions during the dissolution process, thereby decreasing the C-S-H generation and leading to a diminishment in early-age strength [82,84].

However, the effect of the Ca/Si ratio on the late-age compressive strength was related to *N*_c_. When the *N*_c_ varied from 4% to 6%, the trend in late-age strength with varying Ca/Si ratio was consistent with that in early-age strength. When the *N*_c_ further increased to 8%, the late-age strengths of the 0.5 M and 0.25 M rose markedly and surpassed that of the 0.75 M. Therefore, the sequence of late-age strengths was determined as follows: 1.25 M > 1.0 M > 0.25 M > 0.5 M > 0.75 M. The reason for this phenomenon was the extremely high OH^−^ concentration in a solution with 8% *N*_c_, which led to the cleavage of numerous Si-O bonds in 0.5 SP and 0.25 SP, generating substantial quantities of [SiO_4_]^4−^ ions [85]. The increase in [SiO_4_]^4−^ ions extended the chain length of C-S-H gel [25,86] and improved its polymerization degree [87,88,89], thereby strengthening the compactness of the structure [76]. Xue et al. [90] also concluded that an increase in alkali content led to enhanced polymerization degree of the C-S-H gel and improved compressive strength. Additionally, the [SiO_4_]^4−^ ions could polymerize to form silica-rich gel [91], which not only enhanced the connections between the particles, but also filled the voids between the hydration products in the pastes, facilitating a firmer and more unified matrix [92]. Consequently, the late-age compressive strengths of the 0.5 M and 0.25 M were significantly elevated as the *N*_c_ reached 8%.

#### 3.1.4. Optimum Mix Ratio

The compression test results indicated that the highest compressive strengths of AASP materials at 28 days were achieved when the *D*_sc_ was 80% (i.e., activated by a mixture of 80% Na_2_CO_3_ and 20% NaOH) and the *N*_c_ was 8%, with the 28-day strength reaching up to 78.95 MPa, comparable to that of AAS materials. Based on the findings, the optimum mix ratio for Na_2_CO_3_-NaOH based AASP materials was identified as 80% *D*_sc_ and 8% *N*_c_ (C8N2-8).

### 3.2. XRD for Phase Composition Analysis

**Figure 14:**
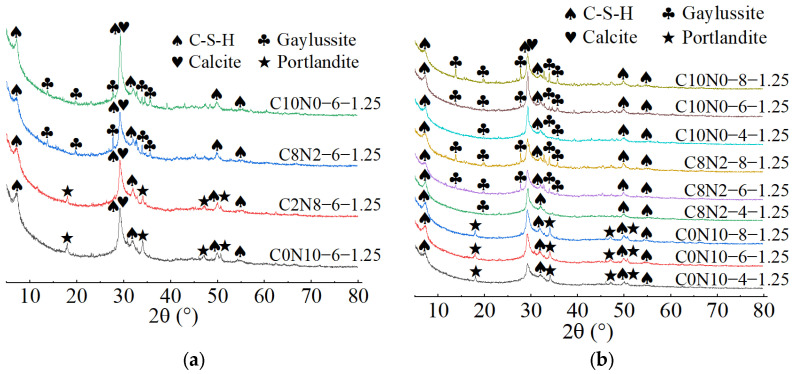
X-ray diffractograms of 1.25 M cured for 28 days: (**a**) changes in the *D*_sc_; (**b**) changes in the *N*_c_. Note: “C8N2-6-1.25” indicates that the precursor is 1.25 SP, the activator is a mixture of 80% Na_2_CO_3_ and 20% NaOH with 6% *N*_c_, and this is similarly applied to other mixes.

**Figure 15:**
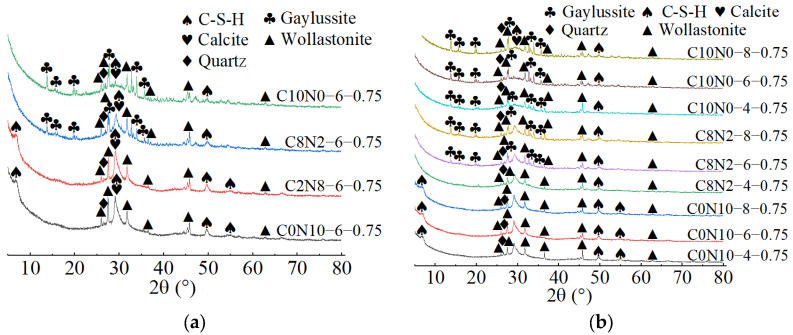
X-ray diffractograms of 0.75 M cured for 28 days: (**a**) changes in the *D*_sc_; (**b**) changes in the *N*_c_.

**Figure 16:**
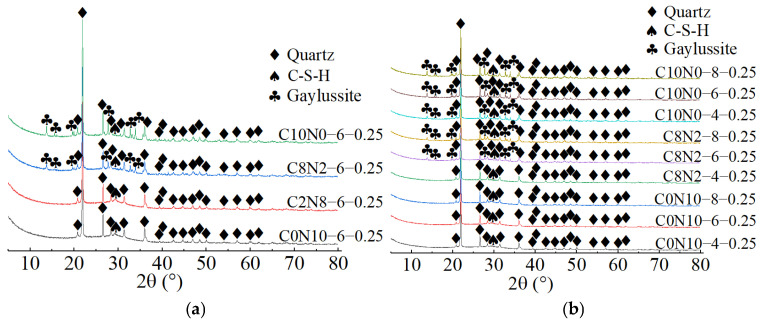
X-ray diffractograms of 0.25 M cured for 28 days: (**a**) changes in the *D*_sc_; (**b**) changes in the *N*_c_.

The X-ray diffractograms of 1.25 M, 0.75 M, and 0.25 M cured for 28 days are depicted in Figure 14, Figure 15 and Figure 16. As indicated in Figure 14a, Figure 15a, Figure 16a, when the *N*_c_ remained constant at 6%, variations in *D*_sc_ led to changes in both the location and number of characteristic peaks, suggesting alterations in the hydration products. As illustrated in Figure 14a, when the *D*_sc_ was 0% and 20%, the hydration products of 1.25 M were calcium silicate hydrate (C-S-H), portlandite (Ca(OH)_2_), and calcite (CaCO_3_). However, when the *D*_sc_ increased to 80% and 100%, the hydration products of 1.25 M were C-S-H, calcite, and gaylussite (Na_2_Ca(CO_3_)_2_·5H_2_O). Portlandite was generated by the reaction of Ca^2+^ with OH^−^ in the alkaline solution; Gaylussite was generated by the reaction of Ca^2+^ with Na^+^ and ${CO}_{3}^{2-}$ in the solution, and calcite was generated by the reaction of Ca^2+^ with ${CO}_{3}^{2-}$ in the solution or CO_2_ in the air [62,65]. Thus, it could be inferred that when the *D*_sc_ was low (0% and 20%), there were few or no ${CO}_{3}^{2-}$ ions in the solution, and Ca^2+^ combined with OH^−^ to form portlandite; when the *D*_sc_ was high (80% and 100%), the OH^−^ concentration decreased, and a significant amount of ${CO}_{3}^{2-}$ ions was present in the solution, which reacted to form calcite and gaylussite.

As demonstrated in Figure 15a, when the *D*_sc_ was 0% and 20%, the hydration products of 0.75 M were C-S-H gel and calcite. The characteristic peaks of C-S-H gel were identified at 6.5°, 29°, 49.5°, and 55° (2θ), of which the main peak located at 29° (2θ) was notably distinct, suggesting that a substantial amount of C-S-H was generated during the reaction. However, when the *D*_sc_ increased to 80% and 100%, the hydration products of 0.75 M were C-S-H gel, calcite, and gaylussite. The diffraction peaks of C-S-H gel were detected at merely 29° and 49.5° (2θ), with the intensity of the main peak gradually decreasing. And, a broad dispersed peak appeared at 29° (2θ) when the *D*_sc_ reached 100%. The following two factors contributed to this: on one hand, the increase in *D*_sc_ reduced the concentration of OH^−^ and the amount of Ca^2+^ dissolved from SP in the solution; on the other hand, Ca^2+^ preferentially reacted with the ${CO}_{3}^{2-}$ and Na^+^ from Na_2_CO_3_ to form calcite and gaylussite [64]. The reduction in Ca^2+^ concentration led to a decrease in C-S-H formation. As presented in Figure 16a, when the *D*_sc_ was 0% and 20%, the hydration product of 0.25 M was C-S-H gel. However, as the *D*_sc_ increased to 80% and 100%, the hydration products of 0.25 M were C-S-H gel and gaylussite. And, the intensity of C-S-H characteristic peak at 29° (2θ) decreased with the increase of *D*_sc_, indicating a reduction in C-S-H generation.

As shown in Figure 14b, Figure 15b, Figure 16b, when the *D*_sc_ remained invariant, changes in *N*_c_ mainly affected the amount of hydration products generated. As indicated in Figure 14b, when the *D*_sc_ was 80% and 100%, the number of gaylussite characteristic peaks detected in 1.25 M with 4% *N*_c_ was fewer than those in the samples with 6% and 8% *N*_c_. The reason could be that the alkalinity of the solution was relatively weak with 4% *N*_c_, leading to fewer Ca^2+^ ions generated from the dissolution of SP. Meanwhile, the amount of Na^+^ and ${CO}_{3}^{2-}$ ions ionized in the activator with 4% *N*_c_ was lower compared to those in the activators with 6% and 8% *N*_c_, resulting in less gaylussite formation. As illustrated in Figure 15b and Figure 16b, when the *D*_sc_ was 80%, no characteristic peaks of gaylussite were detected in 0.75 M and 0.25 M with 4% *N*_c_, while they were observed in the samples with 6% and 8% *N*_c_.

**Figure 17:**
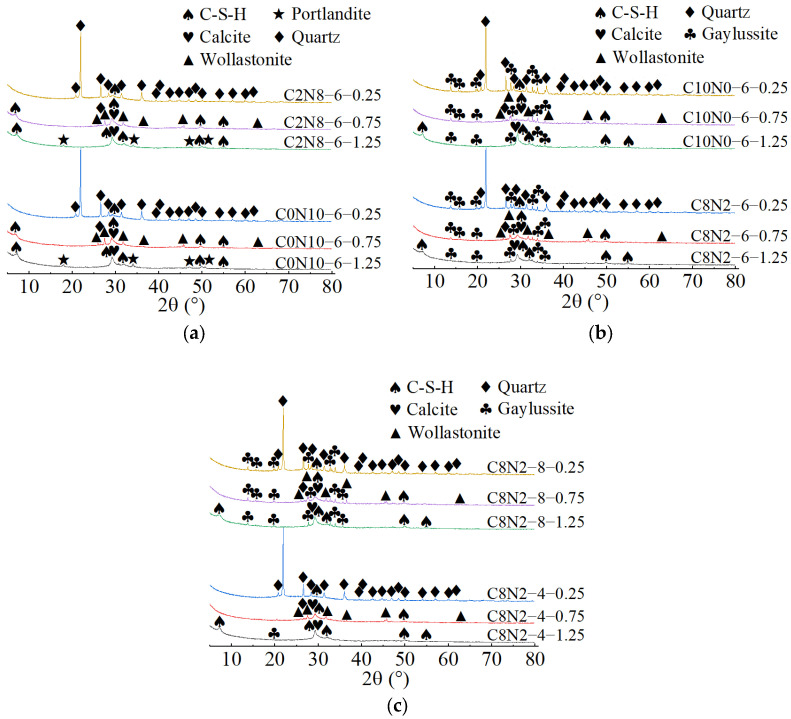
X-ray diffractograms of pastes with different Ca/Si ratios cured for 28 days: (**a**) changes in the *D*_sc_ from 0% to 20%; (**b**) changes in the *D*_sc_ from 80% to 100%; (**c**) changes in the *N*_c_ from 4% to 8%.

When both the *D*_sc_ and *N*_c_ were the same, the X-ray diffractograms of AASP materials cured for 28 days are presented in Figure 17. As indicated in Figure 17a, when the *N*_c_ was 6% and the *D*_sc_ increased from 0% to 20%, the hydration products of all three types of pastes contained C-S-H gel. Additionally, the number and intensity of the C-S-H characteristic peaks gradually decreased as the Ca/Si ratio declined [93]. The C-S-H characteristic peaks were detected at 6.5°, 29°, 32°, 49.5°, and 55° (2θ) in 1.25 M, of which the main peak at 29° (2θ) was remarkably prominent, implying that a considerable amount of C-S-H gel was produced during the reaction. In 0.75 M, the C-S-H gel exhibited diffraction peaks at 6.5°, 29°, 49.5°, and 55° (2θ), with a lower main peak compared to 1.25 M. A subtle characteristic peak of C-S-H gel was observed at 29° (2θ) in 0.25 M, suggesting that the generated C-S-H gel was further decreased. This was attributed to the reduced Ca/Si ratio, which caused a decline in the Ca^2+^ ions generated from the dissolution of SP, thereby decreasing the production of C-S-H gel [84]. However, as the Ca/Si ratio decreased, the main characteristic peak of C-S-H gel at 29° (2θ) gradually broadened, suggesting an enhancement in the polymerization degree of the C-S-H gel [89,94]. Compared to 0.75 M and 0.25 M, portlandite appeared in the hydration products of 1.25 M, resulting from the higher CaO content in 1.25 SP. The dissolved Ca^2+^ ions not only formed C-S-H gel, but also reacted with OH^−^ in the activator to generate portlandite. Compared to 1.25 M and 0.75 M, the X-ray diffractograms of 0.25 M lacked characteristic peaks of calcite, which was attributed to the lower CaO content of the SP used.

As illustrated in Figure 17b, when the *N*_c_ was 6% and the *D*_sc_ rose from 80% to 100%, the hydration products of the pastes included C-S-H gel and gaylussite. Both the quantity and intensity of the C-S-H diffraction peaks progressively diminished with the reduction in the Ca/Si ratio, while the characteristic peaks of gaylussite remained virtually unaffected, resulting from the preferential reaction of Ca^2+^ with ${CO}_{3}^{2-}$ to generate calcite and gaylussite [62]. As depicted in Figure 17c, when the *D*_sc_ was 80% and the *N*_c_ was 8%, gaylussite peaks were identified in all the pastes. However, when the *N*_c_ was 4%, only a weak characteristic peak of gaylussite was observed in 1.25 M, while it was absent in the X-ray diffractograms of 0.75 M and 0.25 M, suggesting that the activator with 4% *N*_c_ was unable to fully activate SP, which aligned with the low strength of specimens with 4% *N*_c_ in mechanical tests.

### 3.3. FT-IR for Chemical Group Analysis

**Figure 18:**
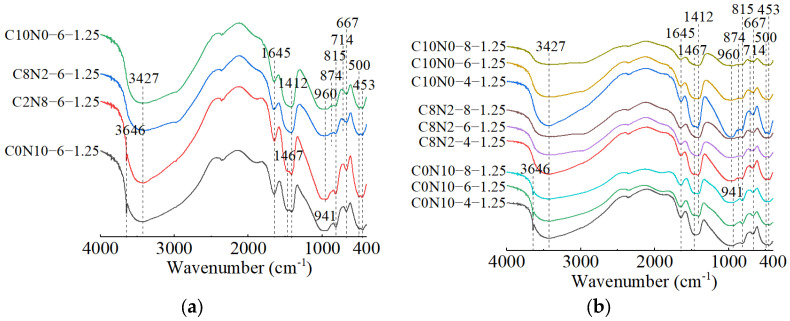
FT-IR spectra of 1.25 M cured for 28 days: (**a**) changes in the *D*_sc_; (**b**) changes in the *N*_c_.

**Figure 19:**
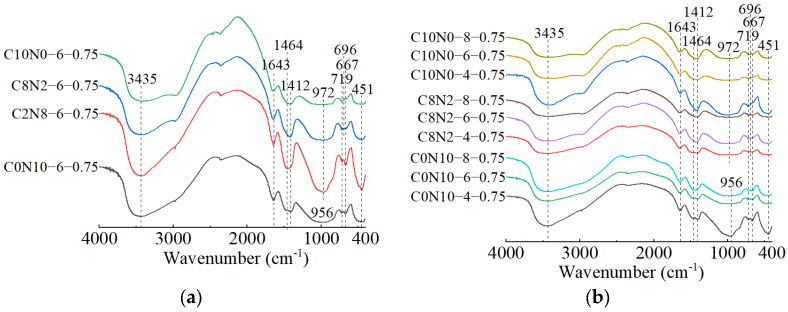
FT-IR spectra of 0.75 M cured for 28 days: (**a**) changes in the *D*_sc_; (**b**) changes in the *N*_c_.

**Figure 20:**
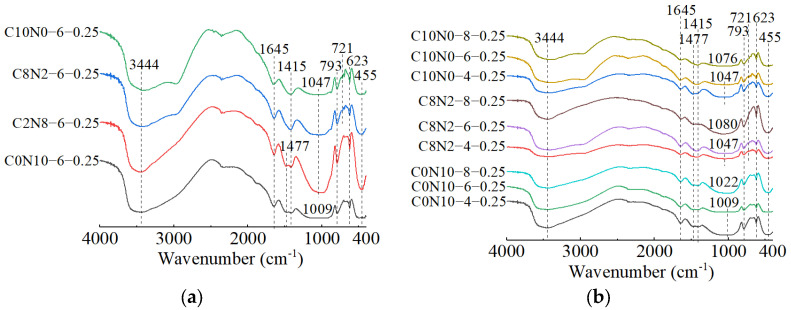
FT-IR spectra of 0.25 M cured for 28 days: (**a**) changes in the *D*_sc_; (**b**) changes in the *N*_c_.

The FT-IR spectra of 1.25 M, 0.75 M, and 0.25 M cured for 28 days are presented in Figure 18, Figure 19 and Figure 20. All of the pastes exhibited some similar bands around 3435, 1645, 1467, 1412, and 453 cm^−1^, revealing the presence of same hydration products [57,63]. The bands at approximately 3435 and 1645 cm^−1^ resulted from stretching and bending vibrations of O-H and H-O-H in H_2_O molecules, respectively [95,96]. The bands at approximately 1467 and 1412 cm^−1^ corresponded to the C-O asymmetric stretching vibrations, suggesting the formation of carbonates [97,98]. The X-ray diffractograms in Section 3.2 identified the existence of calcite and gaylussite in the hydration products of 1.25 M and 0.75 M, which was consistent with the FT-IR analysis. However, characteristic peaks of calcite and gaylussite were not detected in some mix ratios of 0.25 M (e.g., C0N10-4), demonstrating the amount of carbonates generated in 0.25 M was relatively low [98]. The band around 453 cm^−1^ was related to the Si-O in-plane bending vibration caused by the distortion of SiO_4_ tetrahedron [97,99,100], reflecting the cleavage of Si-O bonds during the activating reaction with Na_2_CO_3_-NaOH.

The effects of changes in the *D*_sc_ on the FT-IR spectra of 1.25 M, 0.75 M, and 0.25 M are illustrated in Figure 18a, Figure 19a, Figure 20a. The bands of the 1.25 M around 941 and 960 cm^−1^, the 0.75 M around 956 and 972 cm^−1^, and the 0.25 M around 1009 and 1047 cm^−1^ were all assigned to the Si-O-Si asymmetric stretching vibrations [61,98,101], indicating the C-S-H formation [102]. Meanwhile, when the *D*_sc_ was 0% (i.e., activated by NaOH), the bands at 941 cm^−1^ for 1.25 M, 956 cm^−1^ for 0.75 M, and 1009 cm^−1^ for 0.25 M were observed. These wavenumbers were lower compared to those with other *D*_sc_ values, indicating that the polymerization degree of the C-S-H gel activated by NaOH was lower relative to that activated by Na_2_CO_3_ and a mixture of Na_2_CO_3_ and NaOH [19,61,100], which was consistent with the results of compression tests. When the *D*_sc_ was 0% and 20%, the band at 3646 cm^−1^ in 1.25 M was attributed to the O-H stretching vibration in the Ca(OH)_2_ [29,99], revealing the formation of portlandite, which was aligned with the X-ray diffractograms. When the *D*_sc_ was 80% and 100%, the band at 874 cm^−1^ in 1.25 M was caused by the C-O out-plane bending vibration (υ_2_) [103,104], the bands at 714 cm^−1^ in 1.25 M, 696 cm^−1^ in 0.75 M, and 721 cm^−1^ in 0.25 M were associated with the C-O bending vibration (υ_4_) [103,105], demonstrating more carbonates generated in the reaction.

The effects of changes in the *N*_c_ on the FT-IR spectra of 1.25 M, 0.75 M, and 0.25 M are depicted in Figure 18b, Figure 19b, Figure 20b. As shown in Figure 18b and Figure 19b, changes in the *N*_c_ had minimal effect on the band positions in the FT-IR spectra of 1.25 M and 0.75 M. However, as presented in Figure 20b, when the *N*_c_ reached 8%, the band at 1009 cm^−1^ in 0.25 M shifted to a higher wavenumber at 1022 cm^−1^, and the band at 1047 cm^−1^ shifted to around 1080 cm^−1^, implying the polymerization degree of the C-S-H gel was enhanced [102]. One possible explanation for this was that the 8% *N*_c_ significantly increased the OH^−^ concentration in the solution, resulting in the cleavage of abundant Si-O bonds in 0.25 M and the release of a high quantity of [SiO_4_]^4−^ ions [85], thereby improving the polymerization degree of the C-S-H gel, which matched the mechanical test results. The band at 719 cm^−1^ in 0.75 M stemmed from the C-O bending vibration in the ${CO}_{3}^{2-}$ groups, indicating the presence of calcite or gaylussite [103,105]. The bands located at 667 and 500 cm^−1^ in 1.25 M and 667 cm^−1^ in 0.75 M were representative of Si-O-Si bending vibrations [99,106], associated with the formation of C-S-H gel. The bands around 623 and 793 cm^−1^ in 0.25 M were assigned to Si-O-Si bending vibration and Si-O symmetric stretching vibration [107], respectively, indicating that quartz (SiO_2_) was present in 0.25 M [108,109], in agreement with the XRD analysis.

When the *D*_sc_ and *N*_c_ were the same, a comparison of the main wavebands around 1000 cm^−1^ [110] was performed among the three types of pastes, as shown in Figure 18a, Figure 19a, Figure 20a. The results revealed that as the Ca/Si ratio diminished, the main wavebands shifted from 941 and 960 cm^−1^ for the 1.25 M to 1009 and 1047 cm^−1^ for the 0.25 M. Additionally, the band at 815 cm^−1^ was observed exclusively in 1.25 M, corresponding to the Si-O stretching vibration in the SiO_4_ tetrahedron (Q_1_) [86,99], which indicated the formation of C-S-H gel with a low polymerization degree. Therefore, the decrease of Ca/Si ratio led to a higher polymerization degree of the produced C-S-H gel [111,112], corresponding with the XRD results.

### 3.4. Microstructure and Phase Identification

**Figure 21:**
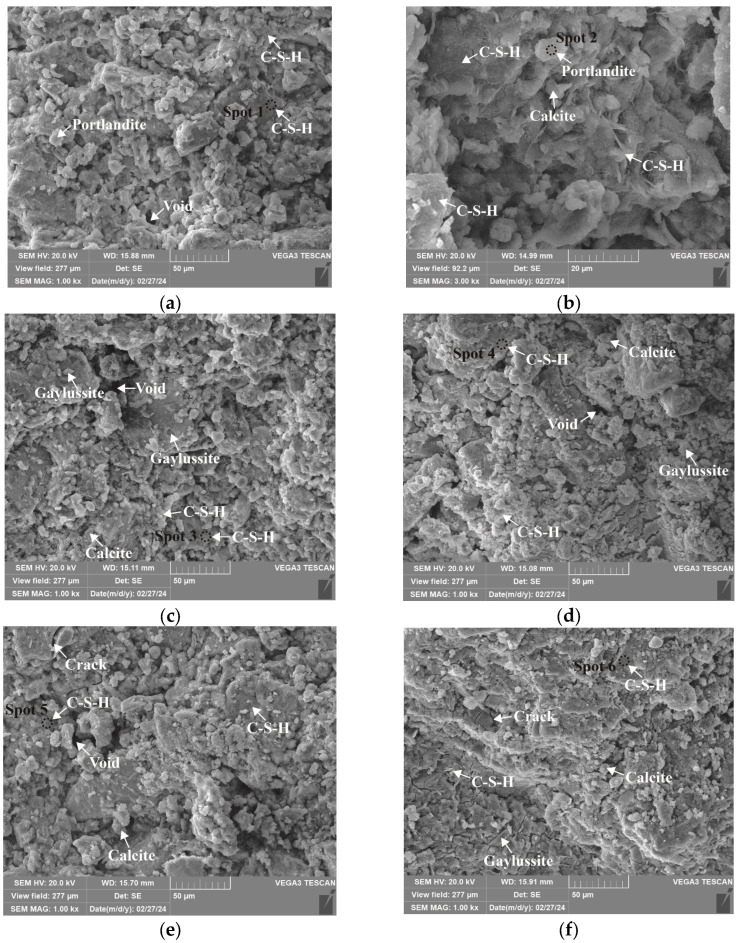
Microstructures of 1.25 M cured for 28 days: (**a**) C0N10-6 (×1k); (**b**) C0N10-6 (×3k); (**c**) C8N2-6 (×1k); (**d**) C10N0-6 (×1k); (**e**) C8N2-4 (×1k); (**f**) C8N2-8 (×1k).

**Figure 22:**
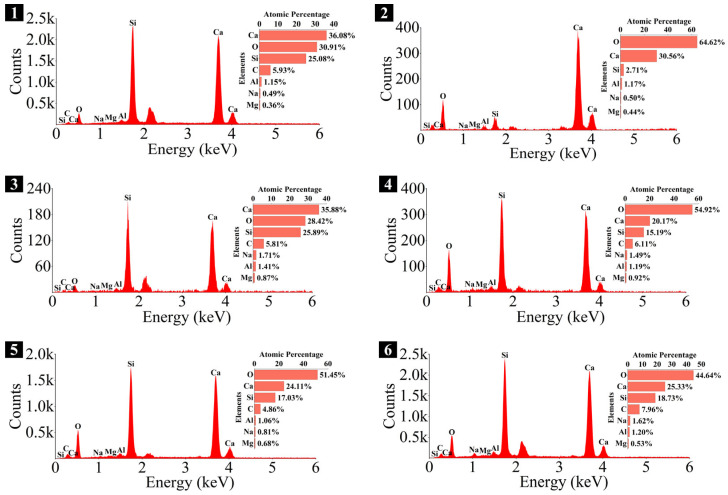
Elemental composition of the spots in 1.25 M.

**Figure 23:**
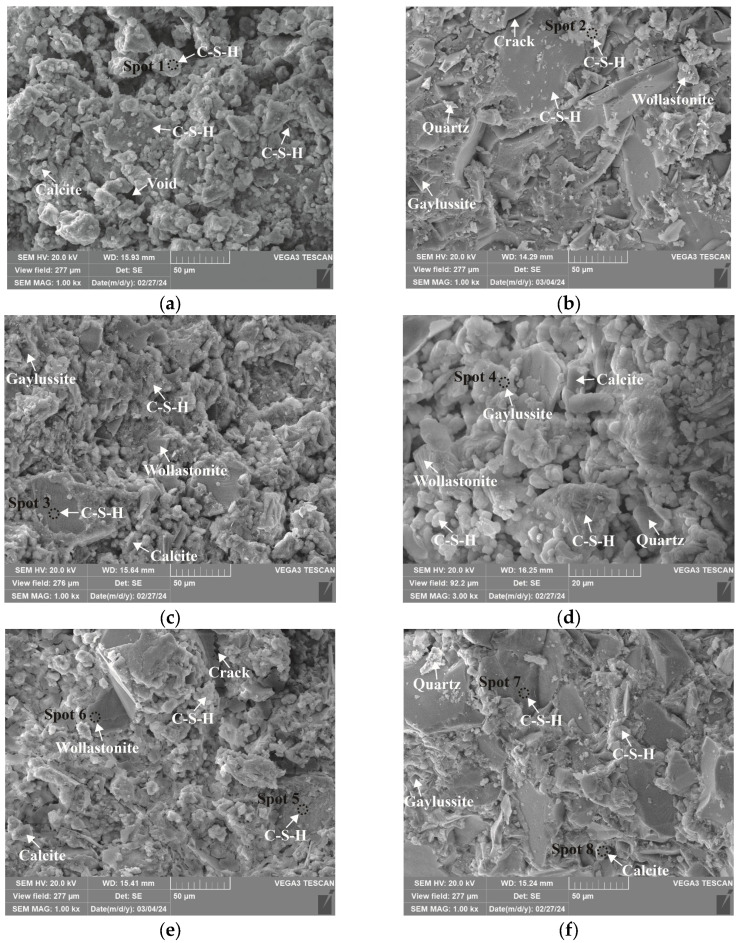
Microstructures of 0.75 M cured for 28 days: (**a**) C0N10-6 (×1k); (**b**) C8N2-6 (×1k); (**c**) C10N0-6 (×1k); (**d**) C10N0-6 (×3k); (**e**) C8N2-4 (×1k); (**f**) C8N2-8 (×1k).

**Figure 24:**
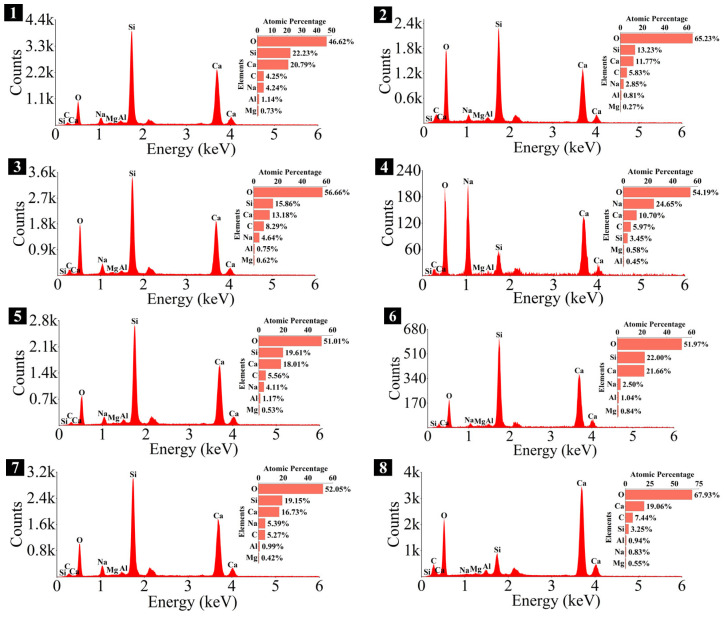
Elemental composition of the spots in 0.75 M.

**Figure 25:**
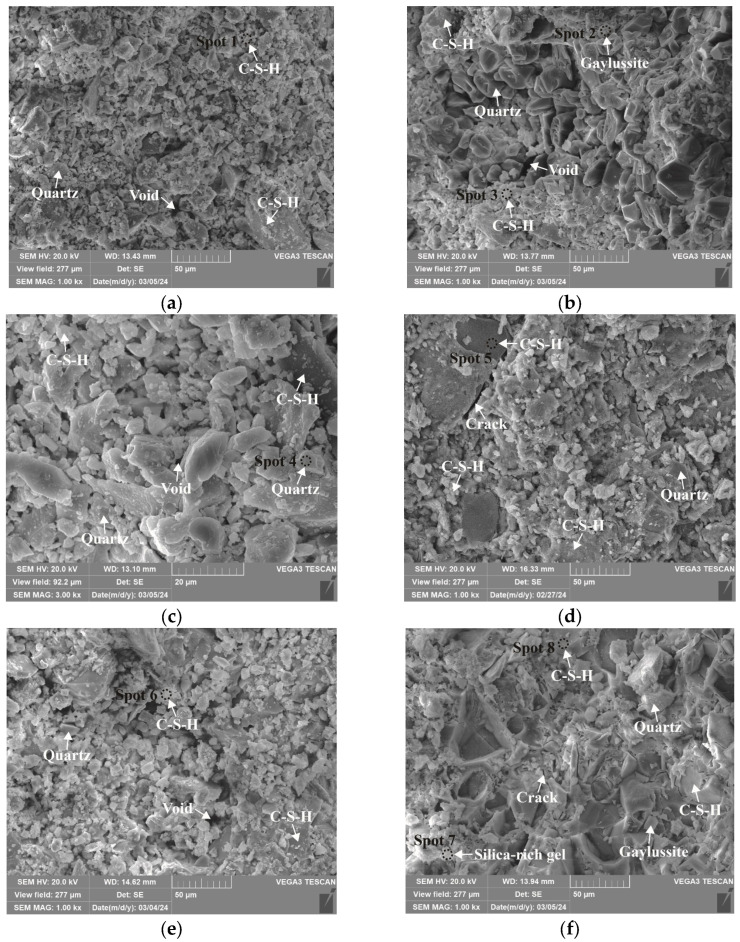
Microstructures of 0.25 M cured for 28 days: (**a**) C0N10-6 (×1k); (**b**) C8N2-6 (×1k); (**c**) C8N2-6 (×3k); (**d**) C10N0-6 (×1k); (**e**) C8N2-4 (×1k); (**f**) C8N2-8 (×1k).

**Figure 26:**
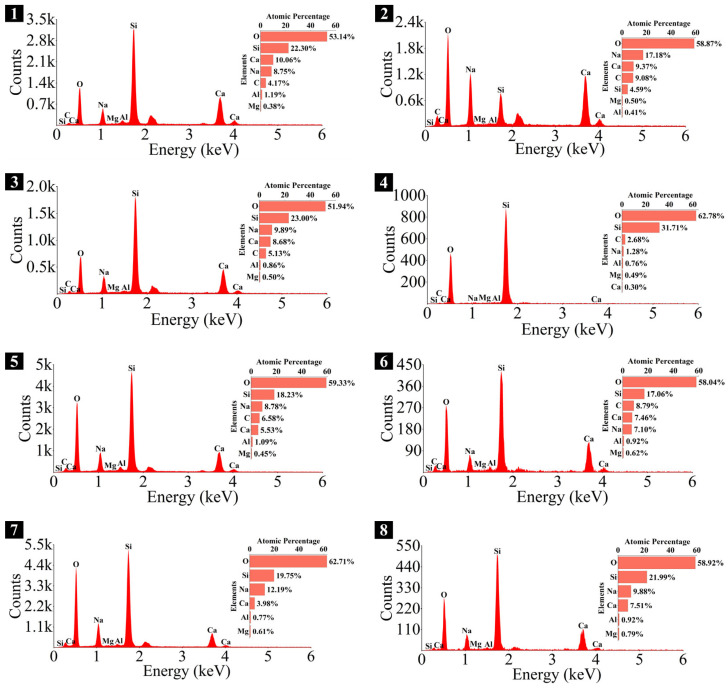
Elemental composition of the spots in 0.25 M.

**Figure 27:**
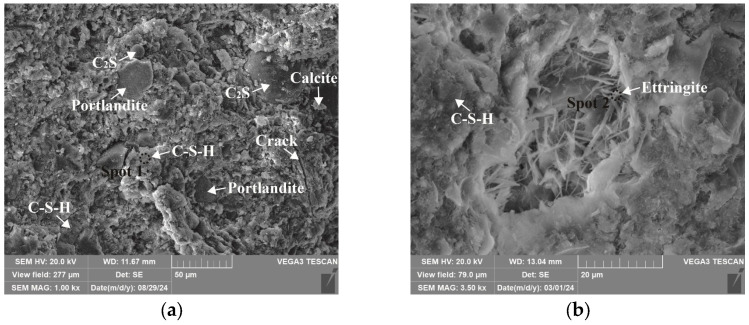
Microstructures of OPC paste cured for 28 days: (**a**) OPC paste (×1k); (**b**) OPC paste (×3.5k).

**Figure 28:**
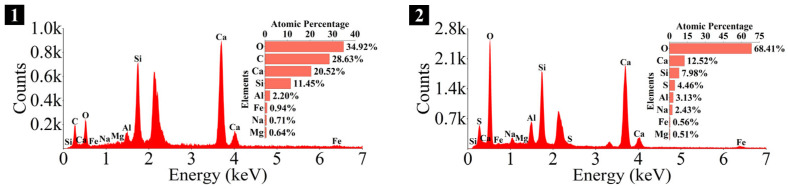
Elemental composition of the spots in OPC paste.

The SEM-EDS analyses of 1.25 M, 0.75 M, and 0.25 M cured for 28 days are presented in Figure 21, Figure 22, Figure 23, Figure 24, Figure 25 and Figure 26, and those for OPC paste are illustrated in Figure 27 and Figure 28. The identified phases are highlighted in the SEM images in light of the microstructural characteristics and EDS analysis. Meanwhile, some EDS spectra are selected and the corresponding atomic ratios are listed in Table 6, Table 7, Table 8 and Table 9.

As depicted in the EDS spectra, O, Ca, Si, and Na were the main elements in the hydration products, demonstrating that C-S-H gel was the primary hydration product of AASP materials. The C-S-H gel in 1.25 M, 0.75 M, and 0.25 M all exhibited a complex structure of interwoven clusters and plates, with small amounts of needle-like and fibrous C-S-H gel observed in some samples. Additionally, C-S-H gel was embedded with different types and shapes of crystal phases: flaky portlandite, along with blocky calcite and gaylussite crystals, were identified in 1.25 M; flaky wollastonite, blocky calcite and gaylussite, along with a small quantity of quartz crystals, were observed in 0.75 M; and a large amount of flaky and blocky quartz, with a few gaylussite crystals, were present in 0.25 M, which was aligned with the X-ray diffractograms and FT-IR spectra. Furthermore, flocculent silica-rich gel was also found attached to the matrix surface of the C8N2-8 sample in 0.25 M (Figure 25f).

As shown in Figure 21a, Figure 23a, and Figure 25a, when the *D*_sc_ was 0% (i.e., activated by NaOH) and the *N*_c_ was 6%, most of the C-S-H gel in the three types of pastes exhibited a cluster-like shape and a loose structure, with poor compactness and numerous void defects, which were associated with the low polymerization degree of the C-S-H gel [61,76]. As presented in Figure 21d, Figure 23c, and Figure 25d, when the *D*_sc_ was 100% (i.e., activated by Na_2_CO_3_) and the *N*_c_ was 6%, the proportion of cluster-like C-S-H gel decreased in the pastes, while the plate-like C-S-H gel increased, resulting in a denser structure. However, the surface remained relatively rough with low flatness, as the ${CO}_{3}^{2-}$ ionized by Na_2_CO_3_ consumed some of the Ca^2+^ ions in the solution, reducing the C-S-H gel formation [64]. As illustrated in Figure 21c, Figure 23b, and Figure 25b, when the *D*_sc_ was 80% (i.e., activated by a mixture of 80% Na_2_CO_3_ and 20% NaOH) and the *N*_c_ was 6%, the matrix was primarily composed of plate-like C-S-H gel, with clustered C-S-H gel filling its pores or adhering to its surface. The matrix structure was uniform and consistent, exhibiting strong integrity and high compactness. This could be attributed to the mixed activation with Na_2_CO_3_ and NaOH, which not only increased the C-S-H production [58], but also enhanced its polymerization degree [61], corresponding to the high compressive strength observed in the tests. When the *D*_sc_ was 80% and the *N*_c_ increased from 4% to 8%, the OH^−^ concentration in the solution rose, which accelerated the hydration reaction and resulted in the large-scale formation of C-S-H gel [82]. Consequently, the matrix exhibited a more compact and uniform microstructure, resulting in an enhancement in compressive strength.

A comparison of the microstructures of 1.25 M, 0.75 M, and 0.25 M revealed that the matrix compactness progressively deteriorated with the decrease in the Ca/Si ratio. Lowering the Ca/Si ratio caused a decline in the CaO content, which resulted in fewer Ca^2+^ ions being released. Consequently, the formation of C-S-H gel was diminished [84], resulting in a rise in defects and porosity in the matrix. However, the silica-rich gel, formed by the abundant [SiO_4_]^4-^ ions released in the solution [91], was observed in the C8N2-8 sample of the 0.25 M, where it contributed to binding the particles and filling the voids [92], making the matrix denser and more homogeneous. As a result, the strength of the C8N2-8 sample in the 0.25 M was higher than that of the 0.75 M with the same mix.

As depicted in Figure 27 and Figure 28, the main elements of the hydration products in the OPC paste were O, Ca, Si, S, and Al, and the identified hydration products included C-S-H gel, flaky portlandite, and needle-like ettringite (Ca_6_Al_2_(SO_4_)_3_(OH)_12_·26H_2_O). The C-S-H gel exhibited a cluster-like shape with a loose structure and numerous cracks, which was attributed to its low polymerization degree [113]. Furthermore, blocky calcite from the raw materials and spherical dicalcium silicate (C_2_S) remaining from the hydration reactions were also observed.

The Ca/Si and Na/Si ratios are two key parameters of C-S-H gel. The Ca/Si ratio determines its polymerization degree [114], while the Na/Si ratio reflects its alkali adsorption capacity [115]. Consequently, the Ca/Si and Na/Si ratios of C-S-H gel with 0% *D*_sc_ (i.e., activated by NaOH) and other *D*_sc_ values (i.e., activated by Na_2_CO_3_ and a mixture of Na_2_CO_3_ and NaOH) are summarized in Table 10 for deeper investigation. It was evident that the Ca/Si ratio of C-S-H gel activated by NaOH was higher, the reason could be that the addition of Na_2_CO_3_ enhanced the ionization of ${CO}_{3}^{2-}$ ions, which preferentially reacted with Ca^2+^ to form insoluble carbonates, such as calcite and gaylussite. As a result, the Ca^2+^ concentration was diminished, leading to a decrease in the Ca/Si ratio of the C-S-H gel [62,69]. In contrast, its polymerization degree was enhanced [89,116], thereby improving the late-age strength of the paste [61,76]. With the same *D*_sc_ and *N*_c_, the Ca/Si ratios for C-S-H in 1.25 M, 0.75 M, and 0.25 M were observed in the range of 1.33–1.44, 0.83–0.94, and 0.30–0.45, respectively, suggesting that a reduction in the Ca/Si ratio of SP led to a decrease in the Ca/Si ratio and an increase in the polymerization degree of the generated C-S-H gel, supported by the XRD and FT-IR results. It was due to the fact that the amount of Ca^2+^ and [SiO_4_]^4−^ ions released was determined by the CaO and SiO_2_ contents of SP, which, in turn, influenced the Ca/Si ratio of the C-S-H gel. Meanwhile, the corresponding Na/Si ratios ranged from 0.02 to 0.10, 0.19 to 0.29, and 0.39 to 0.48, respectively, revealing that the lowered Ca/Si ratio caused an increase in the Na/Si ratio of the C-S-H gel. As depicted in Equation (2), the silanol groups (≡Si-OH) present in the bridging silica converted to ≡Si-O^−^ upon deprotonation in an alkaline solution [25,117], which resulted in the development of negative charges on the surface of the C-S-H gel. However, these negative charges could be neutralized by Na^+^ and Ca^2+^ ions, as shown in Equations (3) and (4), respectively [115]. In general, a higher Ca^2+^ concentration resulted in a lower Na^+^ adsorption by the C-S-H gel, due to the stronger electrostatic interaction of the bivalent Ca^2+^ ions [25,118]. With the reduction in the Ca/Si ratio, the repression of Ca^2+^ was weakened [119], and the deprotonation of silanol groups was increased, which led to a more negatively charged surface of the C-S-H gel [120]. Consequently, more Na^+^ ions were adsorbed, and the Na/Si ratio of the C-S-H gel increased [25,89,121]. Zheng et al. [122] and Feng et al. [123] also proposed that a decrease in the Ca/Si ratio of the C-S-H gel enhanced its alkali adsorption capacity, thereby effectively mitigating the alkali-silica reaction.

≡Si-OH + OH^−^ → ≡Si-O^−^ + H_2_O (2)

≡Si-O^−^ + Na^+^ → ≡Si-ONa (3)

≡Si-O^−^ + Ca^2+^ → ≡Si-OCa^+^ (4)

The Ca/Si and Na/Si ratios for C-S-H in the LSS-activated SP materials and OPC paste are also provided in Table 10 for comparison, with the data for the LSS-activated paste obtained from the previous experiment [78]. Regarding AASP materials, the Ca/Si ratio of the C-S-H gel activated by LSS was the lowest, ranging from 0.22 to 1.28, resulting in a cross-linked structure and the highest polymerization degree [55,61,76,79]; the Ca/Si ratio of the C-S-H gel activated by NaOH was the highest, falling between 0.45 and 1.44, leading to the lowest polymerization degree and a long linear chain structure [61,79]; the Ca/Si ratio of the C-S-H gel activated by Na_2_CO_3_ or a mixture of Na_2_CO_3_ and NaOH fell within the range of the two values mentioned above, yielding a polymerization degree higher than the C-S-H in the NaOH-activated paste and lower than that in the LSS-activated paste [55,61]. The mechanical test results indicated that the compressive strength of the LSS-activated paste was the highest, with the 28-day strength reaching 135.72 MPa, while the NaOH-activated paste exhibited the lowest strength, with the highest strength at 28 days limited to 68.69 MPa, suggesting that the polymerization degree of the C-S-H directly affected the strength [90]. Meanwhile, the conclusion that the Na/Si ratio changed inversely with the Ca/Si ratio of the C-S-H gel was further confirmed: the Na/Si ratio for C-S-H activated by LSS was the highest, with the range of 0.11–0.59, implying the strongest alkali adsorption capacity, while that activated by NaOH was the lowest, ranging from 0.02 to 0.39, demonstrating the weakest alkali adsorption capacity. Additionally, the measured Ca/Si ratio for C-S-H in OPC paste was 1.79, which was in agreement with the findings of relevant studies [97,124,125], where the Ca/Si ratio was reported to fall between 1.5 and 2.0, with an average value of 1.7. The Ca/Si ratio of the C-S-H gel in AASP materials ranged from 0.22 to 1.44, lower than that in OPC paste, revealing a higher polymerization degree of the C-S-H gel in AASP materials [90]. Ravikumar and Neithalath [76], as well as Gruskovnjak et al. [80], also reached the similar conclusions.

## 4. Conclusions

The effects of the Ca/Si ratio in SP, along with the *D*_sc_ and *N*_c_ in the activator, on the strength development of AASP materials were reported in this research. The following key points can be drawn:

(1) Siliceous sand and ground calcium carbonate powder were utilized to produce SP through calcining, water quenching, and grinding, exhibiting latent hydraulic properties similar to those of slag.
(2) The optimum mix ratio for Na_2_CO_3_-NaOH based AASP materials was determined to be 80% *D*_sc_ and 8% *N*_c_ (C8N2-8), with the 28-day strength reaching up to 78.95 MPa, comparable to that of AAS materials.
(3) As the *D*_sc_ increased, the early-age compressive strength and strength development rate of AASP materials gradually decreased. However, the late-age strength rose initially and then decreased, due to the mixed activation with Na_2_CO_3_ and NaOH being more effective than activation by Na_2_CO_3_ or NaOH alone.
(4) The variation in compressive strength with *N*_c_ was influenced by the Ca/Si ratio of SP. When the Ca/Si ratio was 1.25, 1.0, and 0.75, the early-age strengths first rose and then decreased, while the late-age strengths increased steadily with the *N*_c_ increasing from 2% to 8%. However, when the Ca/Si ratio was 0.5 and 0.25, the corresponding pastes showed a continuous increase in both early-age and late-age strengths as *N*_c_ increased from 4% to 8%.
(5) With the decrease in the Ca/Si ratio, the early-age compressive strength diminished accordingly. However, its impact on the late-age compressive strength was related to *N*_c_. When the *N*_c_ ranged from 4% to 6%, the trend in late-age strength mirrored that in early-age strength. As the *N*_c_ further increased to 8%, the late-age strengths of the 0.5 M and 0.25 M surpassed that of the 0.75 M, due to the improved polymerization degree of C-S-H gel and the formation of silica-rich gel.
(6) Microscopic analyses revealed that the primary hydration product of AASP materials was C-S-H gel. With the reduction in the Ca/Si ratio of SP, the generation of C-S-H gel decreased, while its polymerization degree and alkali adsorption capacity increased.

This research utilized locally available materials to prepare SP. Although the activators still need to be transported, the required volume is approximately one-tenth of that for cement [52]. Given that CaO-activated slag binders exhibit excellent durability [48], CaO is particularly well-suited for preparing AAMs in construction applications for remote mountainous regions, as well as islands and reefs far from the inland. Building on this, our forthcoming study aims to prepare CaO as an alkaline activator by calcining local calcareous materials, ultimately overcoming the last obstacle to achieving a self-sufficient solution for construction materials.

## Figures and Tables

**Table 1:** Mix ratios for SP preparation.

Ca/Si Ratio	Siliceous Sand (g)	Ground Calcium Carbonate Powder (g)
1.25	1000	1915
1.0	1000	1531
0.75	1000	1148
0.5	1000	764
0.25	1000	381

Note: The Ca/Si ratio refers to the molar ratio of CaO to SiO_2_.

**Table 2:** Main chemical composition and actual Ca/Si ratios of SP.

Expected Ca/Si Ratio	CaO (%)	SiO_2_ (%)	Al_2_O_3_ (%)	Actual Ca/Si Ratio
1.25	52.41	44.63	1.01	1.258
1.0	46.77	50.47	1.08	0.993
0.75	39.78	57.06	1.13	0.747
0.5	31.10	65.84	1.16	0.506
0.25	18.66	78.39	1.20	0.255

**Table 3:** Mix ratios for Na_2_CO_3_-NaOH based AASP materials (kg/m^3^).

Mix	SP	Na_2_CO_3_	NaOH	Water
C0N10-2	1414.9	0.0	38.0	487.0
C0N10-4 *	1414.9	0.0	76.1	478.8
C0N10-6 *	1414.9	0.0	114.1	470.6
C0N10-8 *	1414.9	0.0	152.1	462.4
C2N8-2	1414.9	7.7	32.0	488.3
C2N8-4 *	1414.9	15.4	64.0	481.4
C2N8-6 *	1414.9	23.0	96.0	474.5
C2N8-8 *	1414.9	30.7	128.0	467.6
C8N2-2	1414.9	36.3	9.5	493.2
C8N2-4 *	1414.9	72.7	18.9	491.1
C8N2-6 *	1414.9	109.0	28.4	489.1
C8N2-8 *	1414.9	145.4	37.9	487.0
C10N0-2	1414.9	48.4	0.0	495.2
C10N0-4 *	1414.9	96.8	0.0	495.2
C10N0-6 *	1414.9	145.1	0.0	495.2
C10N0-8 *	1414.9	193.5	0.0	495.2

Note: “C2N8-4” denotes the *D*_sc_ of 20% and the *N*_c_ of 4%, and similarly for other mixes. “C0N10” and “C10N0” denote activation by NaOH or Na_2_CO_3_ separately. All the mixes were utilized for 1.25 SP, 1.0 SP, and 0.75 SP, while solely the asterisked ones were used for 0.5 SP and 0.25 SP.

**Table 4:** Comparison of strength development in four types of pastes.

Paste	3d	7d	28d
AASP materials	7–29%	13–52%	69–83%
AAS materials [62]	3–19%	26–87%	91–99%
LSS-activated SP materials [78]	16–47%	28–76%	72–95%
OPC paste [78]	42%	68%	78%

Note: “3d” and “7d” indicate the 3-day and 7-day strengths as percentages of the strength at 28 days, respectively. “28d” indicates the 28-day strength as percentage of the strength at 90 days.

**Table 5:** Early-age strength development in pastes with varying *D*_sc_ values.

*D*_sc_	3d	7d
0%	12–29%	23–52%
20%	11–23%	19–40%
80%	8–18%	15–35%
100%	7–18%	13–32%

Note: “3d” and “7d” denote the 3-day and 7-day strengths as percentages of the strength at 28 days, respectively.

**Table 6:** Atomic ratios and phase identifications of the spots in 1.25 M.

Spot	Atomic Ratio	Phase Identification
1	Ca/Si = 1.44, Na/Si = 0.02	C-S-H gel
2	Ca/Si = 11.28, O/Ca = 2.11	Portlandite
3	Ca/Si = 1.39, Na/Si = 0.07	C-S-H gel
4	Ca/Si = 1.33, Na/Si = 0.10	C-S-H gel
5	Ca/Si = 1.42, Na/Si = 0.05	C-S-H gel
6	Ca/Si = 1.35, Na/Si = 0.09	C-S-H gel

**Table 7:** Atomic ratios and phase identifications of the spots in 0.75 M.

Spot	Atomic Ratio	Phase Identification
1	Ca/Si = 0.94, Na/Si = 0.19	C-S-H gel
2	Ca/Si = 0.89, Na/Si = 0.22	C-S-H gel
3	Ca/Si = 0.83, Na/Si = 0.29	C-S-H gel
4	Na/Ca = 2.30, O/Ca = 5.06	Gaylussite
5	Ca/Si = 0.92, Na/Si = 0.21	C-S-H gel
6	Ca/Si = 0.98, O/Ca = 2.40	Wollastonite
7	Ca/Si = 0.87, Na/Si = 0.28	C-S-H gel
8	Ca/Si = 5.86, O/Ca = 3.56	Calcite

**Table 8:** Atomic ratios and phase identifications of the spots in 0.25 M.

Spot	Atomic Ratio	Phase Identification
1	Ca/Si = 0.45, Na/Si = 0.39	C-S-H gel
2	Na/Ca = 1.83, O/Ca = 6.28	Gaylussite
3	Ca/Si = 0.38, Na/Si = 0.43	C-S-H gel
4	Ca/Si = 0.01, O/Si = 1.98	Quartz
5	Ca/Si = 0.30, Na/Si = 0.48	C-S-H gel
6	Ca/Si = 0.44, Na/Si = 0.42	C-S-H gel
7	Na/Si = 0.62, O/Si = 3.18	Silica-rich gel
8	Ca/Si = 0.34, Na/Si = 0.45	C-S-H gel

**Table 9:** Atomic ratios and phase identifications of the spots in OPC paste.

Spot	Atomic Ratio	Phase Identification
1	Ca/Si = 1.79, Na/Si = 0.06	C-S-H gel
2	Ca/Al = 4.00, S/Al = 1.42	Ettringite

**Table 10:** Atomic ratios of the C-S-H gel in AASP materials and OPC paste.

Activator Type		LSS	Na_2_CO_3_ and NaOH	NaOH	OPC
1.25	Ca/Si	1.24–1.28	1.33–1.42	1.44	Ca/Si = 1.79 Na/Si = 0.06
	Na/Si	0.11–0.16	0.05–0.10	0.02	
0.75	Ca/Si	0.71–0.80	0.83–0.92	0.94	
	Na/Si	0.31–0.38	0.21–0.29	0.19	
0.25	Ca/Si	0.22–0.28	0.30–0.44	0.45	
	Na/Si	0.51–0.59	0.42–0.48	0.39	

Note: “Na_2_CO_3_ and NaOH” indicates activation by Na_2_CO_3_ or a mixture of Na_2_CO_3_ and NaOH.

## Data Availability

The original contributions presented in this study are included in the article. Further inquiries can be directed to the corresponding author.

## Abbreviations

The following abbreviations are used in this manuscript:

AASP	Alkali-activated slag-like powder
AAS	Alkali-activated slag
AAMs	Alkali-activated materials
SP	Slag-like powder
1.25 SP	SP with a Ca/Si ratio of 1.25
1.25 M	AASP materials produced from 1.25 SP
MK	Metakaolin
HCFA	Class C fly ash
LCFA	Class F fly ash
OPC	Ordinary Portland cement
*D*_sc_	Dosage of Na_2_CO_3_
*N*_c_	Na_2_O content
LSS	Liquid sodium silicate
C-S-H	Calcium silicate hydrate
XRD	X-ray diffraction
FT-IR	Fourier transform infrared
SEM-EDS	Scanning electron microscopy with energy dispersive spectroscopy
